# Interactive monitoring dashboards for the COVID-19 pandemic in the world anticipating waves of the disease in Brazil with the use of open data

**DOI:** 10.1590/1980-549720240004

**Published:** 2024-02-05

**Authors:** Isaac Negretto Schrarstzhaupt, Marcelo Alves de Souza Bragatte, Letícia Kawano-Dourado, Leonardo Rovatti de Oliveira, Gustavo Fioravanti Vieira, Fredi Alexander Diaz-Quijano, Mellanie Fontes-Dutra

**Affiliations:** I Instituto Capixaba de Ensino, Pesquisa e Inovação em Saúde – Vitória (ES), Brazil.; II Universidade de São Paulo, School of Public Health, Department of Epidemiology, Laboratory of Causal Inference in Epidemiology – São Paulo (SP), Brazil.; III Instituto Mário Schenberg – São Paulo (SP), Brazil.; IV Instituto Todos pela Saúde – São Paulo (SP), Brazil.; V Universidade Federal do Rio Grande do Sul, Department of Genetics, Instituto de Biociências, Laboratory of Immunogenetics, Bioinformatics Center – Porto Alegre (RS), Brazil.; VI Hospital do Coração, Hcor Research Institute – São Paulo (SP), Brazil.; VII Universidade de São Paulo, School of Medicine, Heart Institute, Pulmonology Division – São Paulo (SP), Brazil.; VIII Centro Universitário de Rio Preto, School of Biological Sciences, Department of Biology – São José do Rio Preto (SP), Brazil.; IX Universidade do Vale do Rio dos Sinos, School of Health, Postgraduate Program in Nutrition and Food – Porto Alegre (RS), Brazil.

**Keywords:** Epidemiology, Decision making, COVID-19, Open data research, Epidemiologia, Tomada de decisão, COVID-19, Pesquisa com dados abertos

## Abstract

**Objective:**

Describe the development, implementation, and utilization of dashboards for epidemiological analysis through open data research during the COVID-19 pandemic.

**Methods:**

The dashboards were designed to analyze COVID-19 related public data from various sources, including official government data and social media, at world level. Data processing and cleaning techniques were used to join datasets. We calculated Spearman correlation coefficient between the COVID-like symptoms data of the University of Maryland and Facebook Health research, called COVID Trends and Impacts Survey (CTIS) and the official data of notified COVID-19 cases by the Brazilian Health Ministry.

**Results:**

The dashboards were successful in predicting the onset of new waves of COVID-19 in Brazil. The data analysis revealed a correlation between the CTIS and the official number of cases the country. This article shows the potential of interactive dashboards as a decision-making tool in the context of public health emergencies, as it was used by the official communication of the Rio Grande do Sul state government.

**Conclusion:**

The use of dashboards for predicting the spread of COVID-19 in Brazil was a useful tool for decision-making. To anticipate waves of the disease gives time so that these decisions can be potentially more assertive. This drafts the need of more interdisciplinary actions of this nature, with visualization tools on epidemiologic research.

## RESUMO

**Objetivo:**

Descrever o desenvolvimento, a implementação e o uso de painéis para a análise epidemiológica de dados abertos durante a pandemia de COVID-19.

**Métodos:**

Os painéis foram criados para analisar dados públicos relacionados á COVID-19 de várias fontes, incluindo dados oficiais dos governos e de redes sociais, a nível global. Técnicas de processamento e limpeza foram utilizadas para aglutinar os bancos de dados. Calculamos o coeficiente de correlação de Spearman entre as curvas de sintomas gripais da pesquisa da Universidade de Maryland em conjunto com o Facebook, chamada *COVID Trends and Impacts Survey* (CTIS), e a curva de casos notificados pelo Ministério da Saúde no Brasil.

**Resultados:**

Os painéis obtiveram sucesso em antecipar a chegada de novas ondas de COVID-19 no Brasil. A análise do dado revelou a correlação entre a pesquisa CTIS e o número oficial de casos no país. O artigo destaca o potencial de painéis interativos como uma ferramenta de tomada de decisão no contexto de emergências de saúde pública, como, por exemplo, no uso destes para a comunicação oficial do governo do Rio Grande do Sul.

**Conclusão:**

O uso de painéis para prever o avanço da COVID-19 no Brasil foi uma ferramenta útil para a tomada de decisão. A antecipação de ondas da doença possibilita tempo oportuno para que essas decisões sejam potencialmente mais assertivas. Isso esboça a necessidade de mais ações interdisciplinares dessa natureza, com ferramentas de visualização nas pesquisas epidemiológicas.

## INTRODUCTION

The COVID-19 pandemic highlighted the need to improve the availability and visualization of open data in Brazil so that public health decisions could be made quickly and assertively^1^. Many people use data dashboards, sometimes provided by governments, for decision making^2^. As *SARS-CoV-2* has quickly affected several countries, governments, press professionals, and scientific communicators had to create ways to inform the population in a precise and didactic way, requiring the creation of data dashboards with explanations about the health situation of each municipality, state, and country.

These dashboards have been and still are useful in the current scenario precisely because they standardize different data for visualizing and communicating information^3^. Two successful examples in this context are the Our World in Data website^4^ and the Johns Hopkins University (JHU) dashboard^5^. Both sites focus, in the context of COVID-19, mainly on information on cases and deaths.

With this in mind, *Rede Análise* ^6^, an interdisciplinary collective of researchers and experts, created dashboards with the compilation of multiple open databases from Brazil and the world to increase the assertiveness of decision making. These dashboards contain vaccination coverage, population mobility, hospital data, case and death curves, growth rate, number of people reporting symptoms and the use of masks, in addition to data on Severe Acute Respiratory Syndrome (SARS). All data used in the dashboard is open, that is, available to the general public, which expands research possibilities.

One of the highlights is the COVID-19 Trends and Impact Survey (CTIS), which was carried out jointly by the University of Maryland and Facebook to consult users of the social network on various issues related to the pandemic. The dashboards mentioned in this article used symptom data, where users, if they chose to answer the survey, reported what symptoms they were feeling at that moment. When symptoms of fever, cough, and shortness of breath/difficulty breathing were combined, respondents were considered to be "COVID-like", and this data were then made available for download. This allowed to anticipate the waves of COVID-19 in several Brazilian states from the second wave onward, when tests were carried out more often^7^, helping decision makers based on this anticipation.

The dissemination of information from these dashboards was done using the data storytelling technique, which is a relatively new technique in the area of public health. The concept of data storytelling emerged in 2015^8^, focusing on business professionals, and involves demonstrating technical information in a way that decision makers can reach more assertive conclusions even without specialized technical knowledge.

Visualization favors the transmission of the message, especially when data is abundant and needs to be translated into information for a wide audience. When telling a story, reader or users are guided to reach the information clearly and objectively. Through interactivity, visual dynamism, and emphasis on referenced data, one hopes for successful communication. The objectives of this work were to describe the development and implementation of dashboards for the epidemiological analysis of open data during the COVID-19 pandemic and to evaluate the correlation between trends evidenced from social media with those recorded by the public health surveillance system.

## METHODS

The *Rede Análise* dashboards were created with the Microsoft Power BI tool^9^, powered by public data monitoring the COVID-19^10^ pandemic. The extraction of these data was done in a non-automated way, using the download options via specific websites mentioned below. Five dashboards were created, as shown in Table 1. For counts of cases, deaths, and hospitalizations in different countries, we used the Our World In Data database, which in turn extracts information from the JHU platform^5^.

**Table 1:** List of dashboards created, with link and data source for each one.

Dashboard	Link	Source
Cases, deaths, and growth rate (Brazil and the World)	http://bit.ly/Rede_CasosObitosTaxa	*Our World In Data* (Johns Hopkins University)/Ministry of Health
Vaccination (Brazil)	http://bit.ly/Rede_Vacinas	OpenDataSUS (Ministry of Health)
Hospitalizations (States of RS and SP)	http://bit.ly/Rede_HospitaisRSSP	Fundação SEADE (SP)/RS government
Mobility and Symptoms (Brazil)	http://bit.ly/Rede_MobilidadeSintomas	University of Maryland and Facebook/Google Mobility
SIVEP-GRIPE (Brazilian SARS data)	http://bit.ly/Rede_SIVEPGRIPE	OpenDataSUS (Ministry of Health)

**Figure 1:**
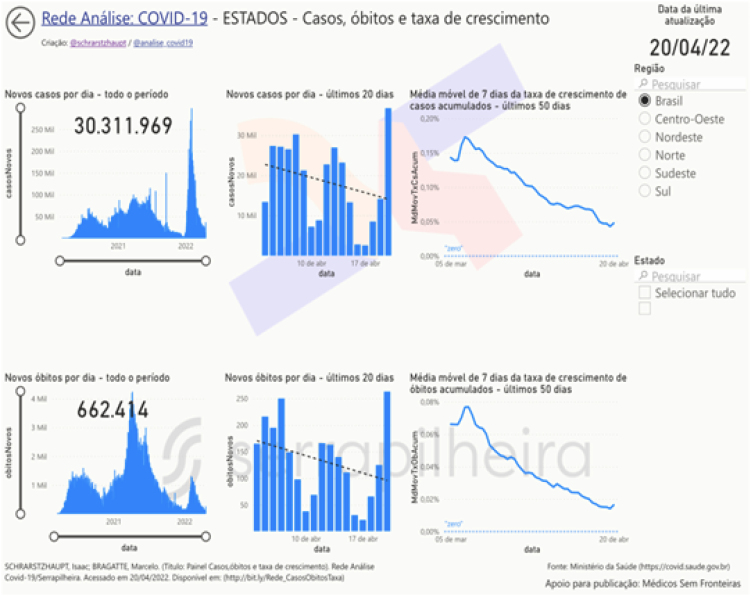
Dashboard of cases, deaths, and growth rate filtered for Brazil as a whole, extracted on 04/20/2022.

For the dashboard of cases and deaths in Brazil (Figure 1), the database used was that of the Ministry of Health^11^. The Seade Foundation database was used for hospitalizations in the state of São Paulo (SP)^12^. For hospitalizations in the state of Rio Grande do Sul (RS), data from the RS State Health Department were used^13^. Only hospitalization data from RS and SP were used, which are available daily and follow the same structure, allowing for historical analysis. For mobility data, the database used was Google^14^. Regarding symptoms and use of masks, we used the CTIS database^15^. For SARS data, we used the Influenza Epidemiological Surveillance System (*Sistema de Vigilância Epidemiológica da Gripe* – SIVEP-GRIPE) database, obtained through OpenDataSUS; finally, for vaccination, data from the National Immunization Plan Information System (*Sistema de Informação do Plano Nacional de Imunizações –* SI-PNI) were used^16–18^.

### Procedures for data extraction

Two types of files were extracted from the Our World In Data database. The first file is *full_data.csv* ^19^ which is obtained from the public domain *github* of Our World in Data, which in turn is powered by JHU. This file contains new cases, new deaths, total cases, total deaths, weekly cases, weekly deaths, fortnightly cases and fortnightly deaths reported by date, as of 01/22/2020, for 216 countries^20^. In addition to countries, there are also regions of interest, such as "World", "Europe", "Low-income countries", among others.

The second file is *owid-covid-data.csv*, which contains, for the same 228 locations, in addition to the data already contained in *full_data.csv*, the data dictionary that can be found in the supplementary material. We highlight the seven-day moving average columns for new cases and deaths and per million inhabitants^21^. This file was extracted from the public domain data on Our World in Data’s github.

The *full_data.csv* file is then imported using the Python programming language, with a script that calculates the growth rates of COVID-19 cases and deaths, among other indicators. The script and field table can be found in the supplementary material. The growth rate formula, used both in cases and deaths, is *x* = (n2/n1) -1, where *x* is the growth rate, *n2* is the number of cases/deaths on a date, and *n1* is the number of cases/deaths from the immediately previous day. This script also calculates the seven-day moving average of new cases reported per day, as well as new deaths reported per day, with the purpose of smoothing the graphs, as notifications drop during weekends and holidays. The moving average of new cases reported per day is calculated by adding the new cases in the last seven days and dividing by seven, generating a new variable in the database, with the same calculation being made with the new deaths reported per day. The *owid-covid-data.csv* source also has data on doses of the COVID-19 vaccine administered per day, in addition to the estimated population of each country. With this, the script also calculates vaccination coverage to make available on the dashboards.

The extraction of data on cases, deaths from Brazil, its states and municipalities is done by transferring a file, obtained from the Ministry of Health’s official COVID-19 monitoring platform^11^. The data are in a compressed file that contains several separate CSV files. Data processing is done by executing a script programmed in python language, which can be found in the supplementary material, as well as in the fields table.

The extraction of hospitalization data from the state of São Paulo is done through the file *plano_sp_leitos_internacoes_serie_nova_variacao_semanal.csv*, without any extra calculations. The extraction of hospitalization data from the state of Rio Grande do Sul is done using the file *transparencia_dados_covid.csv*, without any extra calculations.

Mobility data is extracted using the *Region_Mobility_Report_CSVs.zip* file, which contains 405 files, three files for each of the 135 countries. The three files refer to the years 2020, 2021, and 2022. They are aggregated into one for the countries used in the dashboards (currently Brazil, Chile, Uruguay, United States, Israel, United Kingdom, Indonesia, and India).

The extraction of symptom data is done with a script in python language that fetches the data from the Application Program Interface (API) of the University of Maryland^15,22^. This script allows choosing which indicator to be used, as well as for which states or regions of Brazil. The indicators covid (people who reported feeling COVID-19-like symptoms), flu (people who reported feeling influenza-like symptoms), and mask (people who reported wearing a mask when leaving home) were used. The extraction of SARS data is done through separate files, for the years 2020, 2021, and 2022, without additional calculations. The extraction of vaccination data is done through separate files, one for each state in Brazil^23^.

All files are used as data sources in the Microsoft Power BI Desktop software, to assemble visualization dashboards. These fonts are arranged in all dashboards, in the lower right corner. Power BI was used due to its low implementation cost and also the agility in assembling the dashboards.

It is important to mention that there are typographical errors in database notifications. To emphasize transparency and replicability in the use of data, we chose to keep these errors in the dashboards, explaining them to help educate the population, demonstrating how important it is to keep the database correct and updated. In addition to the educational process of highlighting the need for data curation, maintaining inconsistencies allows analyses to be replicated more reliably by different actors.

These data are presented through data storytelling, with the objective of accelerating understanding on the part of decision makers who, in many cases, do not have similar technical knowledge to those who carried out the analysis and/or synthesis of the information^8^. Thus, it is possible to deliver data both on social networks, to individual decision makers — such as heads of families — and to public and private managers, who need to make their decisions quickly and assertively. This data delivery is constantly updated as data sources are updated, so that decision makers can always search for data dynamically. The script that performs the calculations reported in this section is available on *Rede Análise*’s GitHub^24^.

## RESULTS

**Figure 2:**
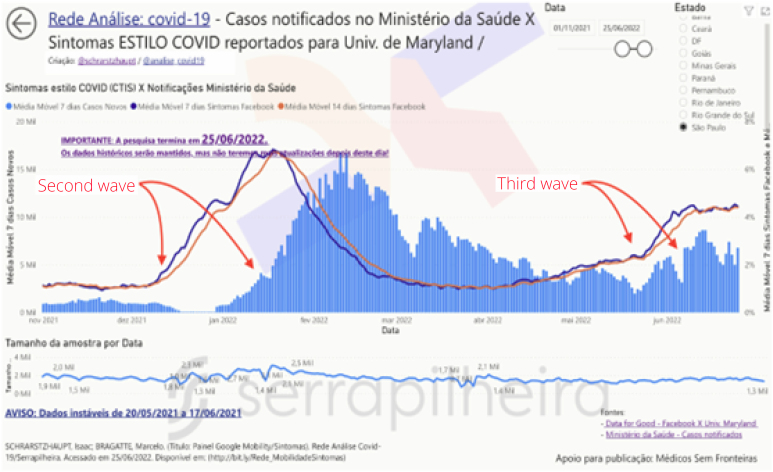
COVID Trends and Impacts Survey dashboard, demonstrating the anticipation of data in the survey in relation to reported cases, here filtered in the state of São Paulo, from 11/01/2021 to 06/25/2022.

**Figure 3:**
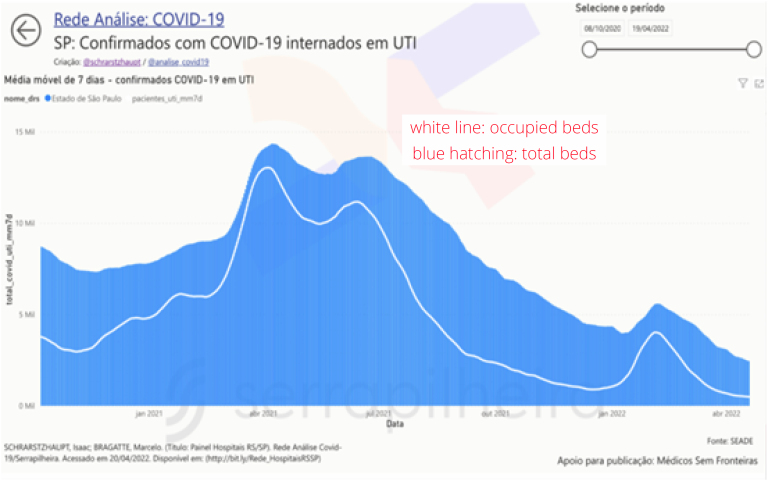
Dashboard of hospitalizations due to COVID-19 in an Intensive Care Unit in the state of São Paulo, demonstrating the use of storytelling to quickly understand how the occupancy of Intensive Care Units changed during the waves of the epidemic.

Mobility and Symptoms dashboards were able to demonstrate in advance the beginning of the second and third waves of COVID-19 in several states in Brazil (Figure 2), through the analysis of data from the CTIS survey. During the first wave, the data were not yet consolidated, with its publication ending in June 2022, making it no longer possible to act in subsequent waves. Furthermore, Hospitalization dashboards also demonstrated the volatility of the ICU occupancy indicator (Figure 3) with the data made available by the state of SP.

The CTIS survey, promoted by the University of Maryland in conjunction with Facebook, worked as follows: users of the social network are invited daily to answer a questionnaire where, among various topics, collects possible symptoms that respondents are feeling at that moment^15^. When symptoms are fever, cough, and shortness of breath/difficulty breathing, respondents are marked as one who may have COVID-19. Dashboards cross-reference this data with case reports by the Ministry of Health in Brazil to show how the research anticipated the rises and falls of the COVID-19 case curve^15^.

This anticipation can be seen when calculating the Spearman correlation coefficient, which was done in a script in the R language, available in the supplementary material. Table 2 presents the correlation coefficients for four Brazilian states that had a significant number of respondents, and in two situations:

**Table 2:** Spearman correlation coefficient between "COVID-19-like" symptom data from the COVID Trends and Impacts Survey and COVID-19 cases reported by the Ministry of Health.

State	Original data dates	CTIS survey data delayed by 20 days
Rio Grande do Sul	0.71902	0.66962
Pernambuco	0.43107	0.82305
Minas Gerais	0.60336	0.92506
São Paulo	0.03000	0.59750

CTIS: COVID Trends and Impacts Survey.

1. Maintaining the original dates, with research demonstrating the increase in cases approximately 20 days before official notifications of COVID-19 cases.
2. Artificially aligning the dates, that is, delaying the research data by 20 days so that it is aligned with official notifications of COVID-19 cases.

Table 2 shows a significant correlation between symptom survey data and official case notifications released by the Ministry of Health.

**Figure 4:**
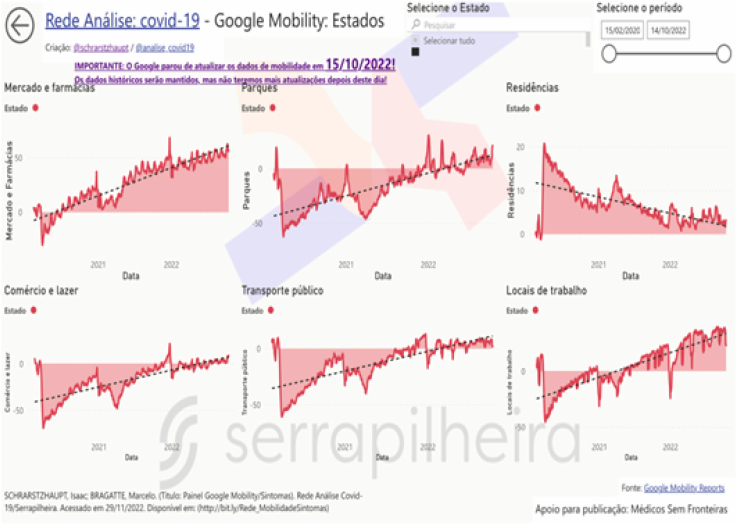
Mobility data dashboard, showing how there were drastic changes in the mobility of the Brazilian population after the start of the COVID-19 pandemic.

In addition to anticipating waves, these dashboards allow seeing and understanding mobility (Figure 4) in Brazil, in its states and municipalities. The dashboard shows data coming from the Community Mobility Reports tool, made available by Google, which, anonymously, collects the number of people visiting six categories of locations: homes, workplaces, public transport stations, parks, markets/pharmacies, and commerce/leisure. These data show how the population’s behavior changed over time during the pandemic^14^.

In addition to these specific data, the dashboards also show data on cases and deaths together with the growth rate of the two indicators, from all states and municipalities in Brazil, in addition to 228 locations worldwide. The data show, in addition to the history of the entire pandemic period, the trend of the last 20 days and the behavior of the growth rate of cases and deaths. All are indicators that, when analyzed together and explained through data storytelling, can assist in the decision-making process^25^.

The dashboards also serve as a window to understand data on SARS cases and deaths reported in SIVEP-GRIPE, with filters by state, municipality, age range, race/color and cause of SARS. This data helps to understand how each age group suffers the impacts of the disease^26^.

The dashboards were made available to the population mainly through threads on the social network Twitter^25^, which reached a total of 74.9 million impressions (views) between January 2021 and January 2022. In May 2021, the dashboards also began to be published in interactive links thanks to the support of Doctors Without Borders. This support allowed the dashboards to be available through reduced links on the *bit.ly* platform, where there were 25,934 engagements with these links. In addition to the dashboards themselves, the storytelling used to demonstrate the data on social networks, in the analyses available on the *Rede Análise* ^27^ website and in live streams is another factor to help in the assertiveness of decisions, as it supports both individual decision makers and public health decision makers^28^.

## DISCUSSION

The use of data dashboards gained notoriety with the COVID-19 pandemic, due to their easy navigation. However, the dashboards themselves cannot explain what is happening and what could happen in current and future scenarios, requiring data analysis/interpretation to extract this information. To ensure easier navigation and increased accessibility, the dashboards were communicated through data storytelling, on social media, with a wide reach of the population and public managers^28^. Furthermore, these dashboards can be accompanied by a "troubleshooting"’ containing frequently asked questions, to reduce the difficulty in understanding the graphics, since this understanding does not only depend on what is in the graph itself, but also on the objective and knowledge of who is seeking the information.

Based on this, these dashboards were created to, through data storytelling, assist in the population’s decision-making, associating social networks in the dissemination of information relevant to the current scenario. With this method, it was possible to visually demonstrate the alert situations that occurred during the pandemic^10,25,29^.

**Figure 5:**
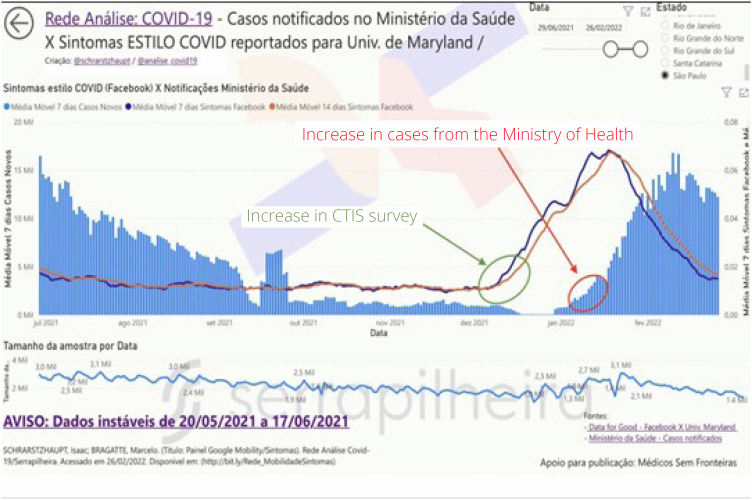
COVID Trends and Impacts Survey dashboard, demonstrating the anticipation of data from individuals with possible COVID-19 according to survey responses in relation to official cases reported by the Ministry of Health, presented on the dashboard through data storytelling.

In addition to the navigation that can be done by anyone, the dissemination of data through the sum of dashboards with the data storytelling technique is of great value to decision makers. In many situations, the data is the responsibility of a technical area, and decision making comes from managers who do not have the same level of knowledge^30^. Through data storytelling, a broader understanding of the data can be achieved by decision makers, who can then be more assertive in their choices. One can see the use of storytelling in communication on social networks (Figure 5) and in its use by decision makers themselves^28^.

Both public managers, citizens, and the press^31^ can use and navigate the dashboards to improve their decision making, which confirms the importance of having an increasingly widespread culture of open data^32^. The state of Rio Grande do Sul used the symptom data dashboard from the CTIS survey as decision-making data due to the anticipation of cases, demonstrated in the methods. As testing has delay problems^33^, the case indicator ends up having a delay in its notification, and indicators that anticipate this wave and are easily accessible, such as in an online dashboard, are of great contribution.

The most acute period of the pandemic brought the public health context closer to the population, and science communication, when becoming a protagonist, needed assertiveness and accuracy in information^34^. Data storytelling appears as an ally, because, by creating analogies together with data dashboards, it is possible to illustrate situations to the point where understanding is achieved more effectively^35^.

There are practical examples of data storytelling in several publications made during the pandemic health emergency^27,36^ where the narrative flow of storytelling is used, which consists of telling the audience what will be demonstrated, in chronological order and, at the end, summarize it, with the most important points and also with the points where there are still gaps in data^8^. A limitation of this work is that we do not have a formal qualitative assessment of dashboard users, such as focal interviews or evaluative questionnaires. However, storytelling received positive support from the feedback from Twitter users, where the threads reached more than 70 million views, a quantitative metric (dependent on audience interaction to generate absolute value) in a defined space of time that reflects the scope of data being transformed into information.

Several similar initiatives have been observed during the COVID-19 pandemic health emergency, both governmental and by volunteer researchers, who played a very important role in disseminating data. In Brazil, there are independent initiatives such as Brasil.Io^37^, which brings data and dashboards, and governmental ones, such as InfoGripe^38^, from Fiocruz, which generated a model to correct the delay in reporting data on hospitalizations due to SARS, which is very important for the decision making. In South Africa, there is the South Africa Medical Research Council (SAMRC)^39^, with the tracking of deaths due to COVID-19, and also initiatives by researchers to understand the excess of deaths from all causes^40,41^.

However, there are still limitations in terms of notification and the availability of higher quality and more frequent public data, there are no data regarding the date of onset of symptoms, date of death, vaccination status, previous infections, among others. We could have them, always respecting the principles of anonymization. This will further improve the creation and use of dashboards; Therefore, storytelling will further assist in individual decision making and in the formulation of public policies.

In the coming years, due to the post-COVID^42^ characteristics and the large number of cases, it is possible that health systems will be pressured by the increase in patients with sequelae arising from this period. Knowing what these consequences are, their frequency and incidence will help both the public authorities to provide funds and the general public to be informed, seeking faster assistance. The democratization of information through constant availability of public data reaches more people and, as it is a free tool for those who use it, it has enormous potential to support public health policies. Dashboards like these tend to be increasingly common, with various applications, such as the monitoring of other respiratory pathogens carried out by *Instituto Todos pela Saúde* ^43^.
