# Supplementary material for: Interactive monitoring dashboards for the COVID-19 pandemic in the world anticipating waves of the disease in Brazil with the use of open data
Source: Rev Bras Epidemiol. 2024 Feb 5;27:e240004. doi: 10.1590/1980-549720240004 (PMC10846413; doi:10.1590/1980-549720240004)
Supplement: Supplementary file 1 [file 1980-5497-rbepid-27-e240004-Suppl01.pdf]

```
#!/usr/bin/env python
# coding: utf-8

# ### notepy_covidbr. v1

# In[1]:

from selenium import webdriver
from selenium.webdriver.common.by import By
from selenium.webdriver.chrome.options import Options


# In[2]:


import pandas as pd
import numpy as np
import glob
import os
import os.path
import patoolib
import shutil
import ctypes


# In[3]:


if os.path.exists("covidbr_rede.csv") == True:
    os.remove("covidbr_rede.csv")
if os.path.exists("full_data_rede.csv") == True:
    os.remove("full_data_rede.csv")


# In[4]:


options = webdriver.ChromeOptions()
download_dir = (os.getcwd()+'\\temp\\br_rar')

options.add_experimental_option("prefs", {
  "download.default_directory": download_dir,
  "download.prompt_for_download": False,
  "download.directory_upgrade": True,
  "safebrowsing.enabled": True
})


# In[5]:


wd_chrome = webdriver.Chrome(options=options)
wd_chrome.get('https://covid.saude.gov.br')
wd_chrome.find_element(By.XPATH, '/html/body/app-root/ion-app/ion-
```

```
router-outlet/app-home/ion-content/div[1]/div[2]/ion-
button').click()


# In[6]:


arqbr_rar = ""
while arqbr_rar == "" :
    arqbr_rar = (','.join(glob.glob(os.getcwd()+'\\temp\\br_rar\
\*.zip')))
wd_chrome.close()
patoolib.extract_archive(arqbr_rar, outdir=(os.getcwd()+'\\temp\
\br_rar'))


# In[7]:


li = []

for arquivo in glob.glob(os.getcwd()+'\\temp\\br_rar\\*.csv'):
    li.append(arquivo)


# In[8]:


# In[9]:


tabelas=[]

for arquivo in li:
    tabelas.append(pd.read_csv(arquivo,index_col=None, header=0,
sep=';', encoding='utf8'))


# In[10]:


covid_br=pd.concat(tabelas, axis=0, ignore_index=True)


# In[11]:


covid_br['municipio'].fillna('',inplace = True)
#display(covid_br)


# In[12]:
```

```
covid_br['data'] = pd.to_datetime(covid_br['data'])


# In[13]:


covid_br.loc[covid_br['municipio'] ==
covid_br['municipio'].shift(1), 'TxCrescCsAcum'] =
covid_br['casosAcumulado'].pct_change()
covid_br['TxCrescCsAcum'].fillna(0,inplace=True)
covid_br['MdMovTxCsAcum'] =
covid_br['TxCrescCsAcum'].rolling(window=7).mean()
covid_br['MdMovTxCsAcum'].fillna(0,inplace=True)

covid_br.loc[covid_br['municipio'] ==
covid_br['municipio'].shift(1), 'TxCrescCs'] =
covid_br['casosNovos'].pct_change()
covid_br['TxCrescCs'].fillna(0,inplace=True)
covid_br['MdMovTxCs'] =
covid_br['TxCrescCs'].rolling(window=7).mean()
covid_br['MdMovTxCs'].fillna(0,inplace=True)

covid_br.loc[covid_br['municipio'] ==
covid_br['municipio'].shift(1), 'TxCrescObAcum'] =
covid_br['obitosAcumulado'].pct_change()
covid_br['TxCrescObAcum'].fillna(0,inplace=True)
covid_br['MdMovTxObAcum'] =
covid_br['TxCrescObAcum'].rolling(window=7).mean()
covid_br['MdMovTxObAcum'].fillna(0,inplace=True)

covid_br.loc[covid_br['municipio'] ==
covid_br['municipio'].shift(1), 'TxCrescOb'] =
covid_br['obitosAcumulado'].pct_change()
covid_br['TxCrescOb'].fillna(0,inplace=True)
covid_br['MdMovTxOb'] =
covid_br['TxCrescOb'].rolling(window=7).mean()
covid_br['MdMovTxOb'].fillna(0,inplace=True)


covid_br['CFR Acum'] = covid_br['obitosAcumulado'] /
covid_br['casosAcumulado']
covid_br['CFR Acum'].fillna(0,inplace=True)

covid_br.loc[:, 'Lockdown'] ="Não"

covid_br['DiaDaSemana'] = (covid_br['data'].dt.weekday)+2

covid_br['MdMovCFRAcum'] = covid_br['CFR
Acum'].rolling(window=7).mean()
covid_br['MdMovCFRAcum'].fillna(0,inplace=True)

covid_br['CFR Diário'] = covid_br['obitosNovos'] /
covid_br['casosNovos']
covid_br['CFR Diário'].fillna(0,inplace=True)
```

```
covid_br['MdMovCFRDia'] = covid_br['CFR
Diário'].rolling(window=7).mean()
covid_br['MdMovCFRDia'].fillna(0,inplace=True)

covid_br['Mortes x Milhao'] = (covid_br['obitosAcumulado'] /
covid_br['populacaoTCU2019'])*1000000

#display(covid_br)


# In[14]:


covid_br.replace(np.inf,0,inplace=True)
covid_br.replace(-np.inf,0,inplace=True)


# In[15]:


url = 'https://github.com/owid/covid-19-data/blob/master/public/
data/owid-covid-data.csv?raw=true'
full_data = pd.read_csv(url, index_col=None, encoding='utf8')
full_data['date'] = pd.to_datetime(full_data['date'])
#display(full_data)


# In[16]:


#full_data.dtypes


# In[17]:


full_data.loc[:, 'Lockdown'] ="Não"

full_data.loc[(full_data['location']=="Germany")|
(full_data['location']=="Austria")|
(full_data['location']=="Belgium")|
(full_data['location']=="Bulgaria")|
(full_data['location']=="Cyprus")|
              (full_data['location']=="Croatia")|
(full_data['location']=="Denmark")|
(full_data['location']=="Slovakia")|
(full_data['location']=="Slovenia")|
(full_data['location']=="Spain")|
              (full_data['location']=="Estonia")|
(full_data['location']=="Finland")|
(full_data['location']=="France")|(full_data['location']=="Greece")|
(full_data['location']=="Hungary")|
              (full_data['location']=="Ireland")|
(full_data['location']=="Italy")|(full_data['location']=="Latvia")|
```

```
(full_data['location']=="Luxembourg")|
(full_data['location']=="Malta")|
              (full_data['location']=="Netherlands")|
(full_data['location']=="Poland")|
(full_data['location']=="Portugal")|
(full_data['location']=="Czechia")|
(full_data['location']=="Romania")|
              (full_data['location']=="Sweden")|
(full_data['location']=="United Kingdom")|
(full_data['location']=="Lithuania"), 'UE'] ="Yes"
full_data['UE'].fillna('No',inplace=True)

full_data.loc[full_data['location'] ==
full_data['location'].shift(1), 'TxCrescCasos'] =
full_data['new_cases'].pct_change()
full_data['TxCrescCasos'].fillna(0,inplace=True)
full_data['MdMovTxCs'] =
full_data['TxCrescCasos'].rolling(window=7).mean()
full_data['MdMovTxCs'].fillna(0,inplace=True)

full_data.loc[full_data['location'] ==
full_data['location'].shift(1), 'TxCrescObitos'] =
full_data['new_deaths'].pct_change()
full_data['TxCrescObitos'].fillna(0,inplace=True)
full_data['MdMovTxOb'] =
full_data['TxCrescObitos'].rolling(window=7).mean()
full_data['MdMovTxOb'].fillna(0,inplace=True)

full_data.loc[full_data['location'] ==
full_data['location'].shift(1), 'TxCrescCsAcum'] =
full_data['total_cases'].pct_change()
full_data['TxCrescCsAcum'].fillna(0,inplace=True)
full_data['MdMovTxCsAcum'] =
full_data['TxCrescCsAcum'].rolling(window=7).mean()
full_data['MdMovTxCsAcum'].fillna(0,inplace=True)

full_data.loc[full_data['location'] ==
full_data['location'].shift(1), 'TxCrscObAcum'] =
full_data['total_deaths'].pct_change()
full_data['TxCrscObAcum'].fillna(0,inplace=True)
full_data['MdMovTxObAcum'] =
full_data['TxCrscObAcum'].rolling(window=7).mean()
full_data['MdMovTxObAcum'].fillna(0,inplace=True)

full_data['Letalidade'] = full_data['total_deaths'] /
full_data['total_cases']
full_data['Letalidade'].fillna(0,inplace=True)

full_data['DiaDaSemana'] = (full_data['date'].dt.weekday)+2

full_data.replace(np.inf,0,inplace=True)
full_data.replace(-np.inf,0,inplace=True)
```

```
# In[18]:


#display(full_data)


# In[19]:


covid_br.to_csv("covidbr_rede.csv")
full_data.to_csv("full_data_rede.csv")


# In[20]:


shutil.rmtree((os.getcwd()+'\\temp'))



# #### Script criado por :
# Leonardo Rovatti, Isaac Schrarstzhaupt, Marcelo Bragatte
```
